# Comparative efficacy of short-term spinal cord stimulation and pulsed radiofrequency in zoster-associated pain: a stratified database study

**DOI:** 10.3389/fneur.2025.1649163

**Published:** 2025-10-22

**Authors:** Fan Lu, Jun Li, Jiwei Zhong, Xuehan Li, Li Song, Ling Ye, Hong Xiao

**Affiliations:** ^1^Department of Pain Management, West China Hospital, Sichuan University, Chengdu, China; ^2^Department of Anesthesia & Operation Center, West China Hospital, Sichuan University, Chengdu, China

**Keywords:** zoster-associated pain, spinal cord stimulation, pulsed radiofrequency, database, stratified analysis

## Abstract

**Background:**

Zoster-associated pain (ZAP) significantly impacts quality of life (QoL) and poses therapeutic challenges. However, there is limited comparative evidence on interventional strategies, particularly regarding short-term spinal cord stimulation (st-SCS) vs. pulsed radiofrequency (PRF), stratified by disease duration and dermatomal involvement.

**Objectives:**

This retrospective study aimed to compare the efficacy and safety of st-SCS and PRF in patients with ZAP, with the primary outcome defined as ≥50% pain reduction at 1 month post-treatment. Secondary outcomes included neuropathic pain characteristics, quality of life (QoL), medication use, and adverse events.

**Methods:**

Clinical data were retrospectively extracted from the institutional pain management database at West China Hospital, covering the period between July 2022 and February 2024. Eligible patients had a clinical diagnosis of ZAP and received either st-SCS or PRF following standard clinical practice. Outcomes assessed included pain severity, neuropathic pain characteristics, QoL indicators, medication usage, and adverse events. Follow-up assessments occurred immediately post-treatment and at 1, 3, 6, and 12 months. Stratified analyses were performed according to disease duration and affected dermatomes.

**Results:**

A total of 186 patients met the inclusion criteria (st-SCS, *n* = 96; PRF, *n* = 90). st-SCS showed superior pain relief compared to PRF, with significantly higher rates of ≥50% pain relief immediately post-treatment (72.92 vs. 14.44%, *P* < 0.001), at 1 month (46.88 vs. 31.11%, *P* = 0.035), and at 3 months (64.58 vs. 43.33%, *P* = 0.005). Stratified analysis indicated greater efficacy of st-SCS in patients with disease durations of 1–2 months and thoracic dermatomal involvement, showing significantly lower NRS scores across multiple follow-ups. Additionally, st-SCS significantly reduced neuropathic pain characteristics, with lower Douleur Neuropathique 4 (DN4) scores at 1 month (1.77 ± 0.80 vs. 2.11 ± 0.99, *P* = 0.046), 3 months (1.42 ± 0.98 vs. 2.29 ± 1.16, *P* < 0.001), and 6 months (1.38 ± 0.93 vs. 1.81 ± 1.02, *P* = 0.008). QoL improvements were consistently greater with st-SCS, particularly regarding sleep quality, mood, and life enjoyment from 1 to 6 months post-treatment.

**Conclusions:**

st-SCS provides superior short-term and sustained pain relief and QoL enhancements compared to PRF in managing ZAP, especially in patients with shorter disease duration and thoracic and abdominal involvement. Both treatments demonstrated comparable safety profiles, confirming the viability and effectiveness of st-SCS as an advantageous interventional option for managing zoster-associated pain.

## 1 Introduction

Zoster-associated pain (ZAP), triggered by the reactivation of varicella-zoster virus (VZV), progresses through distinct phases: acute herpes zoster neuralgia (HZN), subacute herpes zoster (SHZ, occurring 1–3 months post-rash), and postherpetic neuralgia (PHN) if pain persists beyond 3 months (1, 2). The pathophysiology involves peripheral and central sensitization, with sodium channel upregulation, axonal degeneration, and glial activation contributing to chronic pain (3, 4). Globally, approximately 30% of individuals experience HZN in their lifetime, with up to 30% of cases progressing to PHN, particularly in individuals aged over 60 (5). In China, HZN affects 7.7% of the population and imposes significant healthcare and economic burdens (6). ZAP is linked to depression, sleep disturbances, and diminished quality of life (QoL), underscoring the urgent need for effective pain management strategies.

ZAP treatment involves a combination of pharmacologic and interventional approaches. For refractory cases, interventional options such as peripheral nerve blocks, epidural injections, and neuromodulation have gained prominence (7). Among these, pulsed radiofrequency (PRF) has become a minimally invasive alternative, which delivers short bursts of high-frequency electrical current to affected nerves without causing thermal destruction (8). While PRF shows weeks to months efficacy, and it primarily targets peripheral nerves, failing to address central mechanisms (9). Spinal cord stimulation (SCS), a well-established neuromodulation therapy based on the gate control theory, has demonstrated benefits through modulation of pain pathways and glial activity (10, 11). Short-term SCS (st-SCS), involving temporary electrode implantation, has been increasingly applied in ZAP as an interventional strategy, with reports of pain relief and improved quality of life (12).

Although both PRF and SCS are widely used in clinical practice, direct comparative evidence between these two interventions remains scarce, particularly regarding stratified outcomes by disease duration and dermatomal involvement. Previous studies were mostly small-scale and lacked systematic subgroup analysis, limiting the development of clear clinical guidance (13, 14). Therefore, this retrospective study aimed to compare the efficacy and safety of st-SCS vs. PRF in patients with ZAP, with stratified analysis according to disease duration and affected dermatomes.

## 2 Methods

### 2.1 Study design and participants

This retrospective cohort study included patients diagnosed with ZAP who underwent either st-SCS or Pulsed Radiofrequency PRF treatment between July 2022 and February 2024 at the Department of Pain Management, West China Hospital. The study protocol was approved by the institutional ethics committee (Approval No. 2024-2469). The requirement for informed consent was waived. No patient was actively recruited or assigned to treatment groups; patients had received st-SCS or PRF as part of standard clinical practice.

### 2.2 Inclusion and exclusion criteria

Patients were included if they were ≥18 years old, had a clinical diagnosis of ZAP with a pre-treatment Numeric Rating Scale (NRS) score ≥4, and received treatment with either st-SCS or PRF alone. Exclusion criteria included bilateral or multi-site ZAP, chronic pain due to other comorbid conditions, missing key evaluation metrics (pre- and post-treatment pain scores, quality of life assessments, or complication records), and a history of severe psychiatric disorders. Patients were excluded only if incomplete data or new comorbidities precluded valid evaluation.

### 2.3 Procedures

All patients were evaluated by pain specialists using standardized scales, including NRS for pain intensity, the Douleur Neuropathique 4 (DN4) questionnaire for neuropathic pain characteristics, and the Brief Pain Inventory (BPI) for quality of life. For st-SCS, under fluoroscopic guidance, a Tuohy needle was inserted into the epidural space, followed by the implantation of a 1 × 8-contact stimulation electrode (Pins Medical, TL3213), with the lead positioned 1–2 mm lateral to the dorsal column of the spinal cord on the affected side to ensure paresthesia coverage of >50% of the pain area; after securing the lead to the supraspinous ligament, it was connected to a pulse generator (Pins Medical, T902), and short-term stimulation was administered for 2 weeks (1–5 V, 40–100 Hz, 60–500 μs), allowing patients to adjust the intensity as needed. For PRF, under CT guidance, two to three adjacent intervertebral foramina in the corresponding segment were located, and a 21G, 5–15 cm radiofrequency needle was inserted, with sensory testing (< 0.5 V, 50 Hz) performed upon reaching the target to ensure coverage of the pain area; PRF was then administered with parameters set at a temperature of 42 °C, frequency of 2 Hz, pulse width of 20 ms, duration of 600 s, and voltage of 65–100 V for 15 min. All patients underwent two identical PRF sessions during hospitalization, with an interval of 5 days between treatments, which represents the standard protocol in our department.

### 2.4 Data source and collection

All clinical data were retrieved from the institutional pain management database at West China Hospital, which integrates the Hospital Information System and Laboratory Information System. Both the st-SCS and PRF patient groups were derived from this same database, ensuring consistency of data collection and minimizing bias. Trained research assistants collected patient demographics, treatment details, follow-up outcomes, and adverse events. Follow-up data (at pre-treatment, post-procedure, 1, 3, 6, and 12 months) were obtained from existing electronic medical records, including outpatient notes and telephone follow-up records.

### 2.5 Outcomes

The primary outcome was pain intensity, assessed using the NRS, with treatment response defined as ≥50% reduction at 1 month compared with baseline. Secondary outcomes included changes in neuropathic pain characteristics, quality of life, analgesic medication usage, and treatment-related adverse events. These were evaluated at 3, 6, and 12 months when available in the records.

### 2.6 Statistical analysis

Statistical analyses were conducted using SPSS 26.0 software (IBM Corp., Armonk, NY, USA). Continuous data were described using mean and standard deviation (SD), while categorical data were expressed as frequencies and percentages. Between-group comparisons were conducted using independent-samples *t*-tests or Mann–Whitney *U* tests, as appropriate. Categorical data were analyzed using chi-square tests. Repeated measures of NRS and DN4 were analyzed with non-parametric tests and validated with repeated-measures ANOVA to ensure consistency. For baseline variables with significant differences, ANCOVA was performed with adjustment for baseline NRS, disease duration, and age. Missing follow-up values were rare; when present, they were imputed using last observation carried forward (LOCF), and sensitivity analyses confirmed consistency with repeated-measures models. A two-sided *P* value < 0.05 was considered statistically significant.

## 3 Results

### 3.1 Baseline characteristics

**Figure 1:**
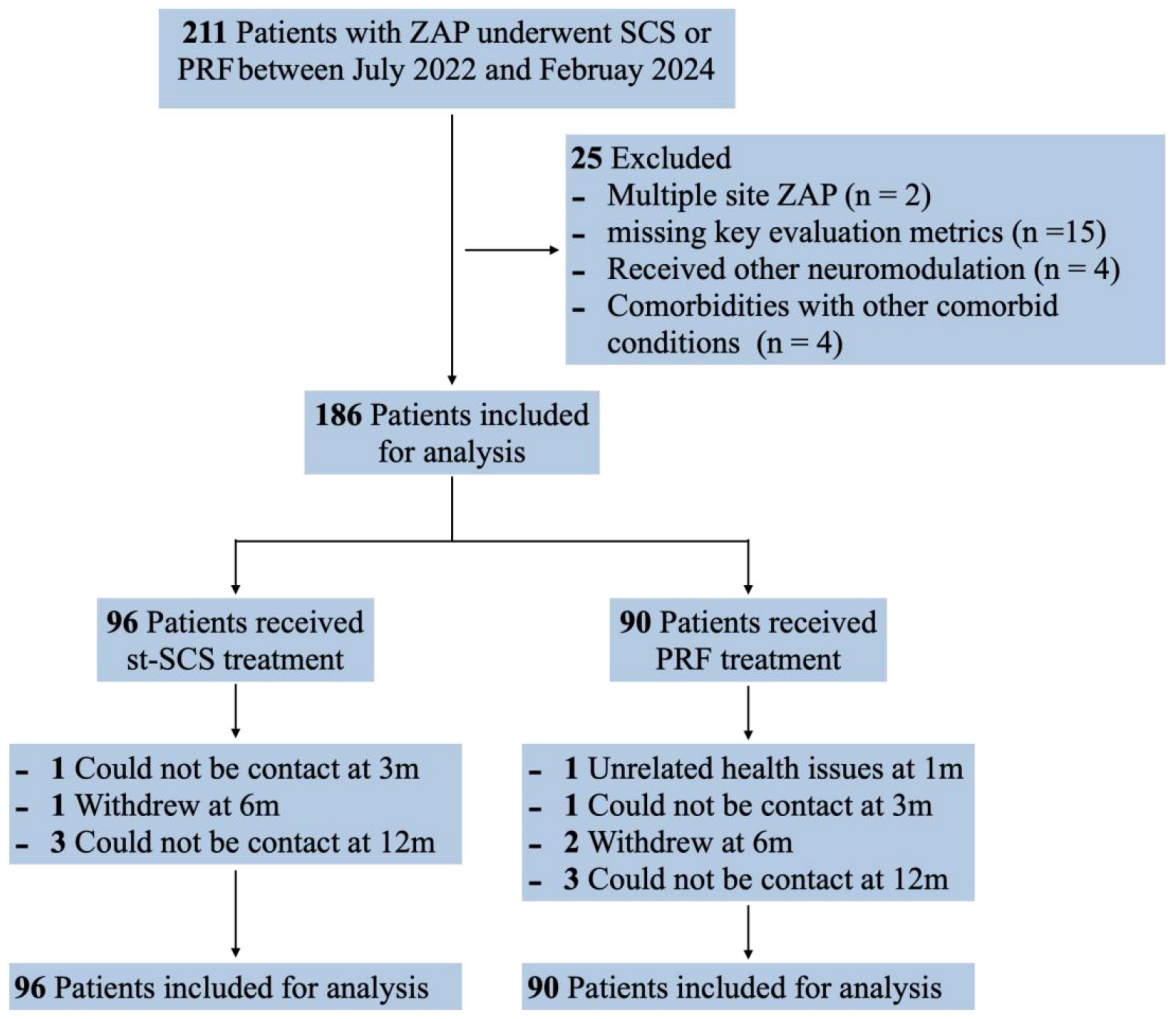
Flowchart of the subject enrollment.

Between July 2022 and February 2024, a cohort of 211 ZAP patients received SCS or PRF intervention, with procedural details systematically documented in the database. Among them, 25 patients were excluded due to eligibility criteria. A total of 186 patients were included in the study, with 96 receiving st-SCS and 90 undergoing PRF. During the 12-month follow-up period, five patients in the st-SCS group and seven patients in the PRF group were lost to follow-up. The primary reasons for loss to follow-up included withdrawal of consent (*n* = 3), health issues (*n* = 1), and inability to contact (*n* = 8; Figure 1). Sensitivity analyses confirmed that the primary outcomes remained consistent, with no significant differences observed between LOCF and complete case analyses (Supplementary Tables S1, S2). Baseline characteristics, including age, gender, disease duration, and affected dermatomes, were comparable between the two groups (Table 1).

**Table 1:** Baseline information.

	**Patients treated with SCS *n* = 96**	**Patients treated with PRF *n* = 90**	***Z*/^2^**	***P* value**
**Age (years)**				
Mean (SD)	65.86 (10.33)	67.74 (9.22)	−1.06	0.289
**Gender**, ***n*** **(%)**				
Male	62 (64.6%)	55 (61.1%)	0.24	0.624^†^
Female	34 (35.4%)	35 (38.9%)		
**Duration of disease (months)**				
Mean (SD)	2.00 (1.15)	2.00 (0.81)	−0.893	0.372
**Duration of disease**, ***n*** **(%)**				
SHZ1: (1 m ≤ and < 2 m)	36 (37.5%)	29 (32.2%)	1.617	0.446^†^
SHZ2: (2 m ≤ and < 3 m)	37 (38.5%)	32 (35.6%)		
PHN: (≥3 m)	23 (24.0%)	29 (32.2%)		
**Pain dermatome**, ***n*** **(%)**				
Neck and upper limbs (C2–8)	14 (14.6%)	30 (33.3%)	9.225	0.026^†^
Thoracic (T1–6)	49 (51.0%)	38 (42.2%)		
Abdomen (T7–12)	21 (21.9%)	14 (15.6%)		
Lumbar and lower limbs (L1–S5)	12 (12.5%)	8 (8.9%)		
**NRS scores**				
Mean (SD)	6.59 (1.22)	6.72 (1.40)	−0.296	0.767
Moderate (4–6)	44 (46.3%)	43 (47.8%)	0.04	0.842^†^
Severe (7–10)	51 (53.7%)	47 (52.2%)		
**DN4 scores**				
Mean (SD)	3.01 (1.01)	3.12 (1.21)	−0.573	0.567
< 3	31 (32.3%)	28 (31.1%)	0.03	0.863^†^
≥3	65 (67.7%)	62 (68.9%)		

^†^Indicates the use of the chi-square test; all others were analyzed using the Mann–Whitney U test.

### 3.2 Pain intensity

**Figure 2:**
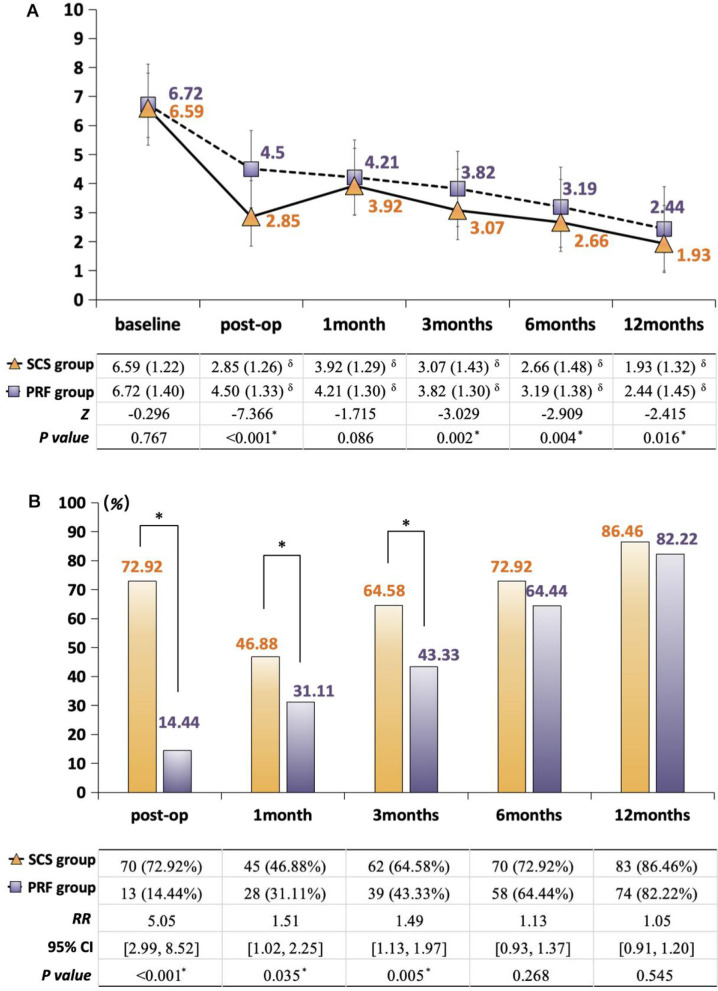
Comparison of pain conditions between the SCS and PRF groups. **(A)** Change in NRS scores, expressed as mean (SD): solid line with yellow triangles: SCS group; dashed line with purple squares: PRF group *indicates significance between SCS and PRF groups (Mann–Whitney *U* test); ^δ^indicates significance between follow-ups and baseline (Wilcoxon signed-rank test); **(B)** Change in pain relief rate, expressed as mean (SD) %: yellow bars: SCS group; purple bars; *indicates significance between SCS and PRF groups (Chi-square test or Fisher's exact test).

Both st-SCS and PRF groups demonstrated significant reductions in NRS scores post-treatment (*P* < 0.05). However, the st-SCS group demonstrated superior pain relief compared to the PRF group at all follow-up time points except for 1 month postoperatively. Notably, at postoperative day 3, the st-SCS group achieved a significantly lower NRS score (2.85 ± 1.26) compared to the PRF group (4.50 ± 1.33, *P* < 0.001). Although there was a slight rebound in NRS scores at 1 month, the st-SCS group maintained a significant reduction in pain compared to the PRF group starting from 3 months (st-SCS: 3.07 ± 1.43 vs. PRF: 3.82 ± 1.30, *P* = 0.002), continuing through 6 months (st-SCS: 2.66 ± 1.48 vs. PRF: 3.19 ± 1.38, *P* = 0.004), and 12 months (st-SCS: 1.93 ± 1.32 vs. PRF: 2.44 ± 1.45, *P* = 0.016; Figure 2A).

### 3.3 Pain relief rate

The proportion of patients achieving ≥50% pain relief was consistently higher in the st-SCS group from the postoperative period up to 3 months. Specifically, at 3 days post-treatment, 72.92% of st-SCS patients achieved ≥50% pain relief, compared to only 14.44% in the PRF group (*P* < 0.001). Similar trends were observed at 1 month (st-SCS: 46.88% vs. PRF: 31.11%, *P* = 0.035) and 3 months (st-SCS: 64.58% vs. PRF: 43.33%, *P* = 0.005). However, no significant differences were observed between the two groups at 6 and 12 months post-treatment (Figure 2B).

### 3.4 Stratified analysis

Patients were stratified into three subgroups based on disease duration: subacute Herpes zoster1 (SHZ1: 1 m ≤ and < 2 m), SHZ2 (2 m ≤ and < 3 m), and postherpetic neuralgia (PHN: ≥3 m). In the SHZ1 subgroup, st-SCS demonstrated significantly lower NRS scores compared to PRF from the postoperative period up to 3 months (*P* < 0.05). Similarly, in the SHZ2 subgroup, st-SCS showed significantly lower NRS scores than PRF from the postoperative period through 3–12 months (*P* < 0.05). In contrast, no significant differences were observed in the PHN subgroup at any follow-up time point except for postoperative day 3 (Table 2).

**Table 2:** Changes in postoperative NRS scores of patients with disease durations.

	**Baseline**	**Post-op**	**1 month**	**3 months**	**6 months**	**12 months**
**SHZ1**						
SCS	6.50 (1.25)	2.72 (1.28)	3.39 (0.99)	2.92 (1.23)	2.56 (1.38)	1.89 (1.19)
PRF	6.69 (1.29)	4.34 (1.11)	4.31 (1.34)	3.86 (1.58)	3.24 (1.55)	2.28 (1.36)
*Z*	−0.387	−4.536	−3.005	−2.676	−1.899	−1.143
*P* value	0.699	< 0.001^*^	0.003^*^	0.007^*^	0.058	0.253
**SHZ2**						
SCS	6.92 (1.04)	2.97 (1.09)	4.14 (1.23)	3.43 (1.59)	2.65 (1.55)	1.57 (1.57)
PRF	7.13 (1.31)	4.69 (1.47)	4.56 (1.16)	4.19 (1.09)	3.47 (1.22)	2.69 (1.69)
*Z*	−0.855	−4.501	−1.385	−2.413	−2.6	−2.711
*P* value	0.392	< 0.001^*^	0.166	0.016^*^	0.009^*^	0.007^*^
**PHN**						
SCS	6.22 (1.35)	2.87 (1.49)	4.39 (1.53)	3.57 (1.41)	2.83 (1.56)	2.57 (0.73)
PRF	6.31 (1.51)	4.45 (1.38)	3.72 (1.31)	3.38 (1.12)	2.83 (1.34)	2.34 (1.26)
*Z*	−0.293	−3.802	−1.62	−0.699	−0.418	−1.107
*P* value	0.769	< 0.001^*^	0.105	0.485	0.676	0.268

NRS scores expressed as mean (SD).

^*^Significance was detected between SCS and PRF group within the same duration; Inter-group comparisons: Mann–Whitney U test.

Patients were further stratified by affected dermatomes: cervical/upper limbs (C2–8), thoracic (T1–6), abdominal (T7–12), and lower limbs (L1–S5). The thoracic subgroup demonstrated the greatest benefit from st-SCS, with significantly lower NRS scores at post-op, 3 months, and 6 months (*P* < 0.05). The abdomen subgroup group also showed improvements favoring st-SCS, particularly at early follow-up time points. In contrast, no significant differences were observed in the cervical/upper limbs and lower limbs subgroups at any follow-up time point except for postoperative day 3 (Table 3).

**Table 3:** Changes in postoperative NRS scores of patients with different dermatomes.

	**Baseline**	**Post-op**	**1 month**	**3 months**	**6 months**	**12 months**
**Cervical and upper limbs (C2–8)**						
SCS	6.64 (1.34)	3.21 (1.05)	3.93 (1.59)	3.93 (1.90)	3.86 (1.88)	2.57 (1.60)
PRF	6.87 (1.31)	4.77 (1.19)	4.47 (1.28)	3.80 (1.10)	3.37 (1.30)	2.47 (1.36)
*Z*	−0.417	−3.617	−1.271	−0.039	−0.791	−0.116
*P* value	0.677	< 0.001^*^	0.204	0.969	0.429	0.907
**Thoracic (T1–6)**						
SCS	6.45 (1.19)	2.67 (1.18)	3.86 (1.21)	3.08 (1.15)	2.41 (1.35)	1.69 (1.19)
PRF	6.63 (1.48)	4.32 (1.47)	4.08 (1.48)	3.74 (1.27)	3.05 (1.37)	2.34 (1.65)
*Z*	−0.163	−4.852	−0.752	−2.575	−2.402	−1.789
*P* value	0.871	< 0.001^*^	0.452	0.010^*^	0.016^*^	0.074
**Abdomen (T7–12)**						
SCS	6.90 (1.26)	2.95 (1.50)	3.76 (1.18)	3.24 (1.61)	2.33 (1.02)	1.86 (1.28)
PRF	6.71 (1.59)	4.57 (1.28)	3.79 (0.80)	3.92 (1.38)	2.57 (1.16)	2.43 (1.28)
*Z*/*t*	−0.346	3.309	−0.127	0.091	−0.666	−1.306
*P* value	0.729	0.002^‡*^	0.899	0.028^‡*^	0.505	0.191
**Lower limbs (L1–S5)**						
SCS	6.58 (1.17)	3.00 (1.35)	4.42 (1.44)	3.33 (1.50)	2.83 (1.59)	2.25 (1.36)
PRF	6.63 (1.19)	4.25 (1.17)	4.63 (1.06)	4.25 (1.28)	4.25 (1.58)	2.88 (1.25)
*t*	0.078	2.139	0.349	2.962	1.959	1.041
*P* value	0.939‡	0.046^‡*^	0.731^‡^	0.058^‡^	0.066^‡^	0.312^‡^

NRS scores expressed as mean (SD).

^*^Significance was detected between SCS and PRF group within the same dermatome.

^‡^Inter-group comparisons using the independent samples t-test; all others were analyzed using the Mann–Whitney U test.

### 3.5 Pain characteristics

Pre-operatively, the most common pain characteristics in both groups were tingling (st-SCS: 80.00%, PRF: 66.67%) and burning pain (st-SCS: 70.83%, PRF: 60.00%; Supplementary Table S3). Postoperatively, the total DN4 scores significantly declined in both groups (*P* < 0.05), with the st-SCS group showing lower scores than the PRF group at 1, 3, and 6 months (*P* < 0.05; Table 4). Burning pain showed greater reduction in the st-SCS group across follow-ups. Numbness temporarily increased in the st-SCS group at 3 days post-treatment but improved significantly by 1 month (Supplementary Table S3). Additionally, the st-SCS group achieved a higher proportion of patients with ≥50% pain area reduction from the postoperative period up to 3 months compared to the PRF group (Supplementary Table S3).

**Table 4:** Comparison of postoperative DN4 scores between the SCS and PRF groups.

	**Baseline**	**Post-op**	**1 month**	**3 months**	**6 months**	**12 months**
SCS group	3.01(1.01)	2.21(1.04)^δ^	1.77(0.80)^δ^	1.42(0.98)^δ^	1.38(0.93)^δ^	1.20(0.88)^δ^
PRF group	3.12(1.21)	1.91(1.10)^δ^	2.11(0.99)^δ^	2.29(1.16)^δ^	1.81(1.02)^δ^	1.01(0.86)^δ^
*Z*	−0.573	−1.928	−1.998	−5.161	−2.634	−1.525
*P* value	0.567	0.054	0.046^*^	< 0.001^*^	0.008^*^	0.127

DN4 scores expressed as mean (SD).

^*^Significance was detected between SCS and PRF group.

^δ^Significance was detected between follow-ups and baseline; Inter-group comparisons: Mann-Whitney U test; intra-group comparisons: Wilcoxon signed-rank test.

### 3.6 Quality of life assessment

**Figure 3:**
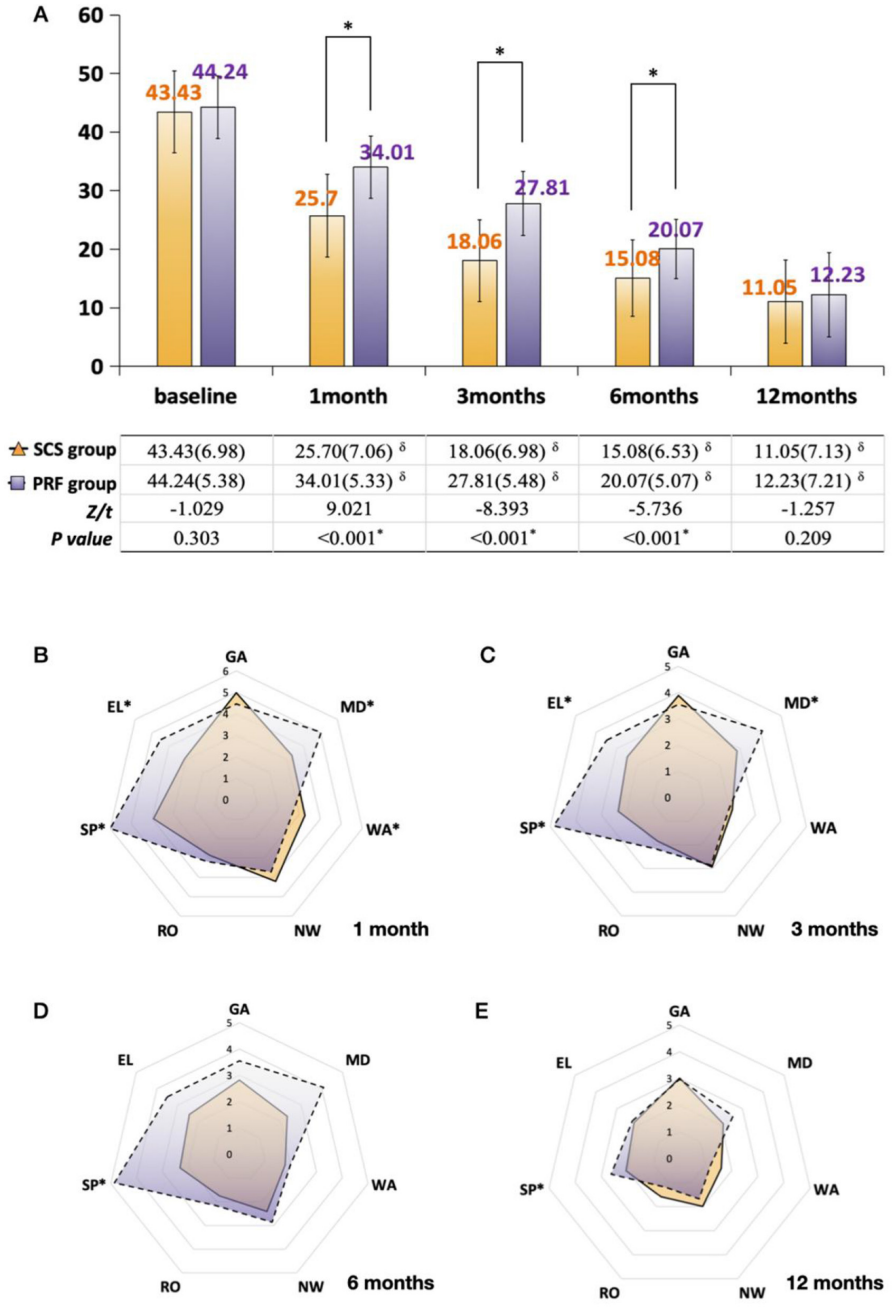
Comparison of BPI pain interference between the SCS and PRF groups. **(A)** Change in total BPI scores, expressed as mean (SD): yellow bars: SCS group; purple bars: PRF group; *indicates significance between SCS and PRF groups (1-month comparison analyzed using independent samples *t*-test; all other inter-group comparisons performed using Mann–Whitney *U* test); ^δ^indicates significance between follow-ups and baseline (Wilcoxon signed-rank test); **(B–E)** Pain interference across seven domains measured by BPI between the SCS and PRF groups: **(B)** domain scores at 1 month; **(C)** domain scores at 3 months; **(D)** domain scores at 6 months; **(E)** domain scores at 12 months. Yellow zone with solid edge: SCS group; purple zone with dashed edge: PRF group; GA, general activity; MD, mood; WA, walking ability; NW, normal work; RO, relationship with others; SP, sleep; EL, enjoyment of life; *indicates significance between SCS and PRF groups.

Both groups showed significant improvements in overall BPI scores after treatment. The st-SCS group demonstrated significantly greater improvements in QoL compared to the PRF group at 1 month (st-SCS: 25.70 ± 7.06 vs. PRF: 34.01 ± 5.33, *P* < 0.001), 3 months (st-SCS: 18.06 ± 6.98 vs. PRF: 27.81 ± 5.48, *P* < 0.001), and 6 months (st-SCS: 15.08 ± 6.53 vs. PRF: 20.07 ± 5.07, *P* < 0.001). By 12 months, both groups achieved similar QoL scores, with no significant difference between them (st-SCS: 11.05 ± 7.13 vs. PRF: 12.23 ± 7.21, *P* = 0.209; Figure 3A). The st-SCS group consistently outperformed the PRF group in mood (MD), sleep (SP), and enjoyment of Life (EL) during the early to mid-term follow-up periods (1–6 months). By 12 months, the differences between groups diminished, with SP remaining the only dimension where st-SCS showed a significant advantage (Figures 3B–E).

### 3.7 Medication usage and adverse events

The most commonly used medications at baseline were Pregabalin (st-SCS: 95.83%; PRF: 93.33%) and Oxycodone Acetaminophen (st-SCS: 35.42%; PRF: 35.56%). Over the 12-month follow-up period, both groups showed significant reductions in medication usage. Overall, the SCS group showed lower medication usage rates across all categories by the 12-month endpoint, although no significant differences were observed between the SCS and PRF groups (Table 5). Common adverse events included transient dizziness, nausea, and local pain at the needle insertion site, with no severe complications reported in either group.

**Table 5:** Changes in medication usage rate between the SCS and PRF groups.

	**Baseline**	**1 month**	**3 months**	**6 months**	**12 months**
**Pregabalin**					
SCS	92 (95.83%)	77 (80.21%)	32 (33.68%)	27 (28.72%)	11 (12.09%)
PRF	84 (93.33%)	68 (76.40%)	31 (35.63%)	24 (27.91%)	13 (15.66%)
**Gabapentin**					
SCS	4 (4.17%)	1 (1.04%)	0 (0.00%)	0 (0.00%)	0 (0.00%)
PRF	5 (5.56%)	0 (0.00%)	0 (0.00%)	0 (0.00%)	0 (0.00%)
**Tramadol and acetaminophen**					
SCS	21 (21.88%)	13 (13.54%)	9 (9.47%)	2 (2.13%)	0 (0.00%)
PRF	17 (18.89%)	9 (10.11%)	7 (8.05%)	1 (1.16%)	1 (1.20%)
**Oxycodone and acetaminophen**					
SCS	34 (35.42%)	28 (29.17%)	14 (14.74%)	9 (9.57%)	7 (7.69%)
PRF	32 (35.56%)	26 (29.21%)	15 (17.24%)	10 (11.63%)	5 (6.02%)
**Tramadol**					
SCS	17 (17.71%)	15 (15.63%)	6 (6.32%)	5 (5.32%)	5 (5.49%)
PRF	15 (16.67%)	10 (11.24%)	3 (3.45%)	3 (3.49%)	3 (3.61%)
**Oxycodone HCl SR tablets**					
SCS	6 (6.25%)	3 (3.13%)	0 (0.00%)	0 (0.00%)	0 (0.00%)
PRF	3 (3.33%)	3 (3.37%)	0 (0.00%)	0 (0.00%)	0 (0.00%)
**Fentanyl patch**					
SCS	4 (4.17%)	0 (0.00%)	0 (0.00%)	0 (0.00%)	0 (0.00%)
PRF	2 (2.22%)	0 (0.00%)	0 (0.00%)	0 (0.00%)	0 (0.00%)

## 4 Discussion

Management of ZAP varies significantly depending on the disease stage. The natural history of ZAP involves a transition from acute HZN to chronic PHN, driven by progressive neuroinflammatory and neuroplastic changes. Studies have shown that early st-SCS significantly improves pain control and sleep quality, potentially preventing PHN progression (15, 16). Additionally, st-SCS outperforms PRF in pain relief at 3 and 6 months in subacute zoster-related pain (17). However, prior studies did not assess how disease duration and zoster-affected dermatomes influence treatment responses. In contrast, our study demonstrates that st-SCS is more effective than PRF modulation in alleviating pain and improving quality of life in patients with ZAP, particularly in the subacute stages of the disease and in patients with thoracic and abdomen involvement. The attenuation of superiority at 12 months is likely attributable to the natural course of pain resolution, the finite persistence of short-term stimulation effects, and reduced statistical power due to attrition.

The superior efficacy of st-SCS in ZAP can be attributed to its multifaceted mechanisms of action. While the gate control theory provides a foundational explanation for its analgesic effects, recent research has highlighted additional mechanisms. Deer et al. demonstrated that SCS modulates neuroinflammation by suppressing glial cell activation and reducing pro-inflammatory cytokine release, which are critical contributors to neuropathic pain (18). In addition, the durability of pain relief improvements observed up to 12 months in our cohort suggests that short-term stimulation may induce lasting neuroplastic changes. This aligns with preclinical evidence showing that spinal cord stimulation modulates dorsal horn neuronal excitability and underlying mechanisms of synaptic plasticity and descending inhibition. For instance, recent studies have demonstrated that SCS robustly activates dorsal horn neurons, suggesting mechanisms of synaptic modulation and enduring circuit-level adaptations (19). Moreover, SCS has also been shown to attenuate dorsal horn neuronal excitability in neuropathic pain models (20). Other evidence suggests that SCS may facilitate nerve repair and remyelination, which are particularly relevant in ZAP given the axonal degeneration and demyelination caused by VZV reactivation (21, 22).These mechanisms likely explain the sustained pain relief observed in our st-SCS group, particularly in patients with early-stage ZAP, where neuroplastic changes are still reversible.

In addition, st-SCS showed notably superior and more sustained effects in patients with thoracic and abdominal ZAP involvement, which is consistent with our previous stratified findings on segmental differences in treatment efficacy (23). This may be anatomically explained by the narrower thoracic epidural space, which ensures closer electrode–cord adherence and more stable stimulation delivery, whereas the wider epidural space at cervical and lumbar levels reduces electrode conformity and increases the risk of lead migration (18). Furthermore, thoracic and abdominal dermatomes are innervated by relatively simple, non-converging segmental nerves, facilitating more precise stimulation coverage. By contrast, limb regions are innervated by complex plexus structures where peripheral mechanisms are more prominent, which may limit SCS efficacy (18). Additionally, the longer nerves in the limbs are more susceptible to axonal degeneration and demyelination, often leading to heightened neuroinflammation in distal regions, potentially reducing neuroplastic responsiveness to SCS in limb regions (24, 25). These mechanisms are anatomically plausible but remain speculative, and future studies are warranted to provide direct mechanistic evidence. For patients with limb-involved ZAP, st-SCS may still be considered by employing dual-lead placement, optimizing stimulation parameters, implementing temporary activity restriction, or combining with peripheral nerve stimulation (26), to improve paresthesia coverage and clinical outcomes.

The st-SCS intervention demonstrated significant improvements across multiple dimensions, most notably in sleep quality. An fMRI study in PHN patients found that 14 days of spinal cord stimulation significantly altered regional homogeneity (ReHo) and degree centrality in key brain regions, including the middle temporal gyrus, parieto-occipital area, superior frontal gyrus, and precentral gyrus, which correlated with improved sleep quality (27). Moreover, the clinical utility of st-SCS is further supported by a significant reduction in medication reliance. By 12 months, only 12.09% of patients in the st-SCS group required pregabalin, and 7.69% required oxycodone/acetaminophen tablets—a statistically significant decrease that reduces the risks associated with long-term pharmacotherapy, such as dizziness, sedation, and gastrointestinal disturbances. Additionally, the safety profile of st-SCS is reinforced by the similar incidence of adverse events observed between the two groups, with no severe complications, motor deficits, or infections reported in the st-SCS cohort.

Furthermore, our stratified findings provide practical guidance for patient selection. Patients in the subacute phase with thoracic or abdominal involvement are optimal candidates for early st-SCS, which yielded superior pain relief and quality-of-life improvements compared with PRF. For limb-involved or chronic PHN cases, st-SCS showed no clear advantage over PRF, but may still be considered by using dual-lead placement, optimized stimulation parameters, temporary activity restriction, or combination with other neuromodulation modalities. In contrast, PRF may serve as an initial, less invasive option for these patients, with escalation to st-SCS if response is inadequate.

## 5 Limitations

This study has several strengths. It is one of the few retrospective analyses to directly compare st-SCS and PRF in ZAP, and the use of a relatively large, hospital-based cohort allowed stratified analysis by disease duration and dermatomes, providing clinically relevant insights. However, several limitations should be acknowledged. First, as a retrospective study, there may be selection bias, incomplete data capture, and recall bias from telephone follow-up. Second, although we attempted to collect comprehensive baseline and outcome variables, potential unmeasured confounders (such as psychosocial status, subtle comorbidities, or patient expectations) might have influenced outcomes but were not available in the database. Third, although stratified analyses reduced heterogeneity, some subgroups had relatively small or uneven sample sizes, particularly in the cervical and upper limb subgroup, which may limit the robustness of comparisons. Finally, our study did not incorporate direct mechanistic data, and the biological explanations discussed remain hypothetical. Future multicenter, prospective randomized studies are warranted to validate these results and further clarify the optimal timing, patient selection, and mechanistic underpinnings of st-SCS therapy.

## 6 Conclusion

Our findings demonstrate that st-SCS is more effective than PRF in alleviating pain and enhancing quality of life for patients with ZAP. The benefits of st-SCS are particularly evident in the subacute stage of the disease and among patients with thoracic and abdominal involvement.

## Data Availability

The original contributions presented in the study are included in the article/supplementary material, further inquiries can be directed to the corresponding author.

## References

[1] Warner BE Goins WF Kramer PR Kinchington PR. A guide to preclinical models of zoster-associated pain and postherpetic neuralgia. Curr Top Microbiol Immunol. (2023) 438:189–221. 10.1007/82_2021_240 34524508 PMC12107716

[2] Yu SY Wan Y Wan Q Ma K Wang JS Lu Z. Chinese expert consensus on the diagnosis and treatment of postherpetic neuralgia. Chinese J Pain Med. (2016) 22:161–7. 10.3969/j.issn.1006-9852.2016.03.001

[3] Bennett GJ Watson CP. Herpes zoster and postherpetic neuralgia: past, present and future. Pain Res Manag. (2009) 14:275–82. 10.1155/2009/380384 19714266 PMC2734513

[4] Yu X Liu H Hamel KA Morvan MG Yu S Leff J. Dorsal root ganglion macrophages contribute to both the initiation and persistence of neuropathic pain. Nat Commun. (2020) 11:264. 10.1038/s41467-019-13839-2 31937758 PMC6959328

[5] Kawai K Gebremeskel BG Acosta CJ. Systematic review of incidence and complications of herpes zoster. BMJ Open. (2014) 4:e004833. 10.1136/bmjopen-2014-004833 PMC4067812 24916088

[6] Liu X Gu L Liu J Hong S Luo Q Wu Y. MRI study of cerebral cortical thickness in patients with herpes zoster and postherpetic neuralgia. J Pain Res. (2022) 15:623–32. 10.2147/JPR.S352105 35250306 PMC8894103

[7] Wang Y Shen Y Guo H You D Jia S Song G. Non-oral pharmacological interventions in the management of herpes zoster-related pain: a review of current research. Front Pain Res. (2024) 5:1485113. 10.3389/fpain.2024.1485113 39664045 PMC11632132

[8] Rui M Ni H Xie K Xu L Yao M. Progress in radiofrequency therapy for zoster-associated pain about parameters, modes, targets, and combined therapy: a narrative review. Pain Ther. (2024) 13:23–32. 10.1007/s40122-023-00561-7 37962817 PMC10796860

[9] Chen L Li J Liu H Yang P Zuo Y Ye L. Interventions for zoster-associated pain: a retrospective study based on the clinical database. Front Neurol. (2022) 13:1056171. 10.3389/fneur.2022.1056171 36504661 PMC9731217

[10] Garcia K Wray JK Kumar S. Spinal cord stimulation. In: StatPearls [Internet]. Treasure Island, FL: StatPearls Publishing (2025). Available online at: https://www.ncbi.nlm.nih.gov/books/NBK553154/31985947

[11] Caylor J Reddy R Yin S Cui C Huang M Huang C. Spinal cord stimulation in chronic pain: evidence and theory for mechanisms of action. Bioelectron Med. (2019) 5:12. 10.1186/s42234-019-0023-1 31435499 PMC6703564

[12] Zuo L Su A Xie Y Yang X. Clinical study of short-term spinal cord stimulation for herpes zoster-associated pain. Eur J Med Res. (2024) 29:603. 10.1186/s40001-024-02196-6 39702473 PMC11657820

[13] Shanthanna H Eldabe S Provenzano DA Bouche B Buchser E Chadwick R. Evidence-based consensus guidelines on patient selection and trial stimulation for spinal cord stimulation therapy for chronic non-cancer pain. Reg Anesth Pain Med. (2023) 48:273–87. 10.1136/rapm-2022-104097 37001888 PMC10370290

[14] Atkinson L Sundaraj SR Brooker C O'Callaghan J Teddy P Salmon J. Recommendations for patient selection in spinal cord stimulation. J Clin Neurosci. (2011) 18:1295–302. 10.1016/j.jocn.2011.02.025 21719293

[15] Huang J Yang S Yang J Sun W Jiang C Zhou J. Early treatment with temporary spinal cord stimulation effectively prevents development of postherpetic neuralgia. Pain Physician. (2020) 23:E219–30. 10.36076/ppj.2020/23/E219 32214307

[16] Dong DS Yu X Wan CF Liu Y Zhao L Xi Q. Efficacy of short-term spinal cord stimulation in acute/subacute zoster-related pain: a retrospective study. Pain Physician. (2017) 20:E633–45. 10.1097/MD.0000000000029073 28727708

[17] Wan CF Song T. Efficacy of pulsed radiofrequency or short-term spinal cord stimulation for acute/subacute zoster-related pain: a randomized, double-blinded, controlled trial. Pain Physician. (2021) 24:215–22. 10.36076/ppj.2021/24/215 33988940

[18] Deer TR Mekhail N Provenzano D Pope J Krames E Thomson S. The appropriate use of neurostimulation: avoidance and treatment of complications of neurostimulation therapies for the treatment of chronic pain. Neuromodulation. (2014) 17:571–97. 10.1111/ner.12206 25112891

[19] Wang D Lee KY Kagan ZB Bradley K Lee D. Frequency-dependent neural modulation of dorsal horn neurons by kilohertz spinal cord stimulation in rats. Biomedicines. (2024) 12:1346. 10.3390/biomedicines12061346 38927553 PMC11201430

[20] Guan Y Wacnik PW Yang F Carteret AF Chung CY Meyer RA. Spinal cord stimulation-induced analgesia: electrical stimulation of dorsal column and dorsal roots attenuates dorsal horn neuronal excitability in neuropathic rats. Anesthesiology. (2010) 113:1392–405. 10.1097/ALN.0b013e3181fcd95c 21068658

[21] Zhang H Zhang Z Lin H. Research progress on the reduced neural repair ability of aging Schwann cells. Front Cell Neurosci. (2023) 17:1228282. 10.3389/fncel.2023.1228282 37545880 PMC10398339

[22] Mahalingam R Feia B Coleman C Anupindi K Saravanan P Luthens A. Simian varicella virus pathogenesis in skin during varicella and zoster. Viruses. (2022) 14:1167. 10.3390/v14061167 35746639 PMC9227806

[23] Lu F Zhong J Liu H Xiao H. CT-guided paravertebral injection of doxorubicin for treatment of postherpetic neuralgia: a database-based retrospective stratified study. Front Neurol. (2023) 14:1258464. 10.3389/fneur.2023.1258464 37767531 PMC10520975

[24] Höke A. Mechanisms of disease: what factors limit the success of peripheral nerve regeneration in humans? Nat Clin Pract Neurol. (2006) 2:448–54. 10.1038/ncpneuro0262 16932603

[25] Calvo M Dawes JM Bennett DL. The role of the immune system in the generation of neuropathic pain. Lancet Neurol. (2012) 11:629–42. 10.1016/S1474-4422(12)70134-5 22710756

[26] Slangen R Schaper NC Faber CG Joosten EA Dirksen CD van Dongen RT. Spinal cord stimulation and pain relief in painful diabetic peripheral neuropathy: a prospective two-center randomized controlled trial. Diabetes Care. (2014) 37:3016–24. 10.2337/dc14-0684 25216508

[27] Fan X Ren H Bu C Lu Z Wei Y Xu F. Alterations in local activity and functional connectivity in patients with postherpetic neuralgia after short-term spinal cord stimulation. Front Mol Neurosci. (2022) 15:938280. 10.3389/fnmol.2022.938280 36034501 PMC9405669

