# Supplementary material for: Comparative efficacy of short-term spinal cord stimulation and pulsed radiofrequency in zoster-associated pain: a stratified database study
Source: Front Neurol. 2025 Oct 22;16:1649163. doi: 10.3389/fneur.2025.1649163 (PMC12586037; doi:10.3389/fneur.2025.1649163)
Supplement: Supplementary file 1 [file Table_1.doc]

| **Supplemental Table 1. Comparison of ITT (LOCF) and Complete Case Analysis for NRS Scores Over Time** | | | | | | |
| --- | --- | --- | --- | --- | --- | --- |
| **Time Point** | **Group** | **ITT (LOCF)  (Mean ± SD)** | **CC (Mean ± SD)** | **ITT Sample Size** | **CC Sample Size** | **P value** |
| baseline | SCS | 6.59 (1.22) | 6.59 (1.22) | 96 | 96 | >0.99 |
|  | PRF | 6.72 (1.40) | 6.72 (1.40) | 90 | 90 | >0.99 |
| post-op | SCS | 2.85 (1.26) | 2.85 (1.26) | 96 | 96 | >0.99 |
|  | PRF | 4.50 (1.33) | 4.50 (1.33) | 90 | 90 | >0.99 |
| 1 month | SCS | 3.92 (1.29) | 3.92 (1.29) | 96 | 96 | >0.99 |
|  | PRF | 4.21 (1.30) | 4.23 (1.23) | 90 | 89 | >0.99 |
| 3 months | SCS | 3.07 (1.43) | 3.06 (1.35) | 96 | 95 | >0.99 |
|  | PRF | 3.82 (1.30) | 3.85 (1.32) | 90 | 87 | >0.99 |
| 6 months | SCS | 2.66 (1.48) | 2.68 (1.39) | 96 | 94 | >0.99 |
|  | PRF | 3.19 (1.38) | 3.18 (1.42) | 90 | 86 | >0.99 |
| 12 months | SCS | 1.93 (1.32) | 1.88 (1.25) | 96 | 91 | >0.99 |
|  | PRF | 2.44 (1.45) | 2.39 (1.37) | 90 | 83 | >0.99 |
| NRS scores expressed as mean (SD); ITT: Intention-to-Treat; LOCF: Last Observation Carried Forward; CC: Complete Case; NRS: Numerical Rating Scale. | | | | | | |
|  |  |  |  |  |  |  |
