# Supplementary material for: Comparative efficacy of short-term spinal cord stimulation and pulsed radiofrequency in zoster-associated pain: a stratified database study
Source: Front Neurol. 2025 Oct 22;16:1649163. doi: 10.3389/fneur.2025.1649163 (PMC12586037; doi:10.3389/fneur.2025.1649163)
Supplement: Supplementary file 2 [file Table_2.doc]

| **Supplemental Table 2. Comparison of ITT (LOCF) and Complete Case Analysis for DN4 Scores Over Time** | | | | | | |
| --- | --- | --- | --- | --- | --- | --- |
| **Time Point** | **Group** | **ITT (LOCF)  (Mean ± SD)** | **CC (Mean ± SD)** | **ITT Sample Size** | **CC Sample Size** | ***P value*** |
| baseline | SCS | 3.01 (1.01) | 3.01 (1.01) | 96 | 96 | >0.99 |
|  | PRF | 3.12 (1.21) | 3.12 (1.21) | 90 | 90 | >0.99 |
| post-op | SCS | 2.21 (1.04) | 2.21 (1.04) | 96 | 96 | >0.99 |
|  | PRF | 1.91 (1.10) | 1.91 (1.10) | 90 | 90 | >0.99 |
| 1 month | SCS | 1.77 (0.80) | 1.77 (0.80) | 96 | 96 | >0.99 |
|  | PRF | 2.11 (0.99) | 2.03 (0.78) | 90 | 89 | >0.99 |
| 3 months | SCS | 1.42 (0.98) | 1.43 (0.76) | 96 | 95 | >0.99 |
|  | PRF | 2.29 (1.16) | 2.29 (1.21) | 90 | 87 | >0.99 |
| 6 months | SCS | 1.38 (0.93) | 1.40 (1.01) | 96 | 94 | >0.99 |
|  | PRF | 1.81 (1.02) | 1.83 (0.98) | 90 | 86 | >0.99 |
| 12 months | SCS | 1.20 (0.88) | 1.22 (0.91) | 96 | 91 | >0.99 |
|  | PRF | 1.01 (0.86) | 1.07 (0.97) | 90 | 83 | >0.99 |
| DN4 scores expressed as mean (SD); ITT: Intention-to-Treat; LOCF: Last Observation Carried Forward; CC: Complete Case; DN4: Douleur Neuropathique 4. | | | | | | |
|  |  |  |  |  |  |  |
