# Supplementary material for: Comparative efficacy of short-term spinal cord stimulation and pulsed radiofrequency in zoster-associated pain: a stratified database study
Source: Front Neurol. 2025 Oct 22;16:1649163. doi: 10.3389/fneur.2025.1649163 (PMC12586037; doi:10.3389/fneur.2025.1649163)
Supplement: Supplementary file 3 [file Table_3.doc]

| **Supplemental Table 3. Pain Characteristics of Patients between the SCS and PRF Groups** | | | | | | | |
| --- | --- | --- | --- | --- | --- | --- | --- |
|  |  | **baseline** | **post-op** | **1month** | **3months** | **6months** | **12months** |
| **Pain characteristics** | | | | | | | |
| Burning pain | SCS | 68 (70.83%) | 16 (16.17%) | 23 (23.96%) | 4 (4.21%) | 3 (3.19%) | 3 (3.30%) |
|  | PRF | 54 (60.00%) | 16 (17.78%) | 15 (16.85%) | 11 (12.64%) | 8 (9.30%) | 7 (8.54%) |
| Cold pain | SCS | 4 (4.17%) | 0 (0.00%) | 0 (0.00%) | 1 (1.05%) | 0 (0.00%) | 0 (0.00%) |
|  | PRF | 3 (3.33%) | 3 (3.33%) | 3 (3.37%) | 3 (3.45%) | 0 (0.00%) | 0 (0.00%) |
| Electric shock pain | SCS | 50 (52.08%) | 12 (12.50%) | 8 (8.33%) | 11 (11.58%) | 7 (7.45%) | 5 (5.49%) |
|  | PRF | 42 (46.67%) | 26 (28.89%) | 15 (16.85%) | 15 (17.24%) | 12 (13.95%) | 12 (14.63%) |
| Tingling | SCS | 78 (81.25%) | 36 (37.50%) | 15 (15.63%) | 6 (6.32%) | 6 (6.38%) | 7 (7.69%) |
|  | PRF | 72(80.00%) | 42 (46.67%) | 25 (20.09%) | 16 (18.39%) | 11 (12.79%) | 6 (7.32%) |
| Pins and needles | SCS | 64 (66.67%) | 42 (43.75%) | 21 (21.88%) | 25 (26.32%) | 15 (15.96%) | 12 (13.19%) |
|  | PRF | 57 (63.33%) | 25 (27.78%) | 21 (23.60%) | 25 (28.74%) | 25 (29.07%) | 23 (28.05%) |
| Numbness | SCS | 31 (32.29%) | 92 (95.83%) | 14 (14.85%) | 12 (12.63%) | 12 (12.77%) | 10 (10.99%) |
|  | PRF | 21 (23.33%) | 17 (18.89%) | 14 (15.73%) | 18 (20.69%) | 19 (22.09%) | 19 (23.17%) |
| Itching | SCS | 21 (21.88%) | 7 (7.29%) | 23 (23.96%) | 8 (8.42%) | 3 (3.19%) | 0 (0.00%) |
|  | PRF | 11 (12.22%) | 7 (7.78%) | 7 (7.87%) | 8 (9.20%) | 8 (9.30%) | 8 (9.76%) |
| Hypoesthesia to touch | SCS | 58 (60.41%) | 14 (14.58%) | 13 (13.54%) | 13 (113.68%) | 7 (7.45%) | 7 (7.67) |
|  | PRF | 50 (55.56%) | 24 (26.67%) | 20 (22.47%) | 13 (14.94%) | 10 (111.63%) | 3 (3.66%) |
| Hypoesthesia to pinprick | SCS | 58 (60.42%) | 6 (6.25%) | 8 (8.33%) | 6 (6.32%) | 6 (6.38%) | 6 (6.59%) |
|  | PRF | 32 (35.56%) | 17 (18.89%) | 16 (17.98%) | 10 (11.49%) | 5 (5.81%) | 3 (3.66%) |
| Provoked by brushing | SCS | 55 (57.29%) | 18 (18.757%) | 26 (27.08%) | 15 (15.76%) | 9 (9.57%) | 7 (7.69%) |
|  | PRF | 38 42.22%) | 28 (31.11%) | 26 (29.21%) | 11 (12.64%) | 9 (10.47%) | 4 (4.88%) |
| **Pain area** | | | | | | | |
| Expansion | SCS | / | 2 (2.08%) | 3 (3.13%) | 1 (1.05%) | 0 (0.00%) | 0 (0.00%) |
|  | PRF | / | 2 (2.22%) | 1 (1.12%) | 2 (2.30%) | 0 (0.00%) | 0 (0.00%) |
| Invariant | SCS | / | 10 (10.42%) | 6 (6.25%) | 5 (5.26%) | 8 (8.51%) | 6 (6.59%) |
|  | PRF | / | 20 (22.22%) | 16 (17.98%) | 15 (17.24%) | 7 (8.14%) | 7 (8.54%) |
| Reduction <50% | SCS | / | 21 (21.88%) | 20 (20.83%) | 17 (17.89%) | 13 (13.83%) | 8 (8.79%) |
|  | PRF | / | 25 (27.78%) | 27 (30.34%) | 21 (21.14%) | 21 (24.42%) | 8 (9.76%) |
| Reduction  ≥ 50% | SCS | / | 63 (65.63%) | 67 (69.79%) | 69 (72.63%) | 73 (77.66%) | 77 (84.62%) |
|  | PRF | / | 43 (47.78%) | 45 (50.56%) | 49 (56.35%) | 58 (67.44%) | 67 (81.71%) |
