# Supplementary material for: Comparative efficacy of short-term spinal cord stimulation and pulsed radiofrequency in zoster-associated pain: a stratified database study
Source: Front Neurol. 2025 Oct 22;16:1649163. doi: 10.3389/fneur.2025.1649163 (PMC12586037; doi:10.3389/fneur.2025.1649163)
Supplement: Supplementary file 4 [file Table_4.doc]

| **Supplemental Table 4. Subgroup sample sizes across follow-up intervals** | | | | | | | |
| --- | --- | --- | --- | --- | --- | --- | --- |
|  |  | **baseline** | **post-op** | **1month** | **3months** | **6months** | **12months** |
| **SCS** | **Total** | 96 | 96 | 96 | 95 | 94 | 91 |
| **PRF** | **Total** | 90 | 90 | 89 | 88 | 86 | 83 |
|  |  |  |  |  |  |  |  |
| **SCS** | **Duration of Disease** |  |  |  |  |  |  |
|  | SHZ1: (1m ≤ and ＜2 m) | 36 | 36 | 36 | 36 | 35 | 35 |
|  | SHZ2: (2m ≤ and ＜3 m) | 37 | 37 | 37 | 37 | 37 | 35 |
|  | PHN: (≥3 m) | 23 | 23 | 23 | 22 | 22 | 21 |
|  |  |  |  |  |  |  |  |
| **PRF** | **Duration of Disease** |  |  |  |  |  |  |
|  | SHZ1: (1m ≤ and ＜2 m) | 29 | 29 | 29 | 29 | 29 | 29 |
|  | SHZ2: (2m ≤ and ＜3 m) | 32 | 32 | 32 | 31 | 31 | 30 |
|  | PHN: (≥3 m) | 29 | 29 | 28 | 28 | 26 | 24 |
|  |  |  |  |  |  |  |  |
| **SCS** | **Pain dermatome** |  |  |  |  |  |  |
|  | Neck and upper limbs (C2-8) | 14 | 14 | 14 | 14 | 14 | 13 |
|  | Thoracic (T1-6) | 49 | 49 | 49 | 48 | 47 | 46 |
|  | Abdomen (T7-12) | 21 | 21 | 21 | 21 | 21 | 21 |
|  | Lumbar and lower limbs (L1-S5) | 12 | 12 | 12 | 12 | 12 | 11 |
|  |  |  |  |  |  |  |  |
| **PRF** | **Pain dermatome** |  |  |  |  |  |  |
|  | Neck and upper limbs (C2-8) | 30 | 30 | 30 | 30 | 29 | 29 |
|  | Thoracic (T1-6) | 38 | 38 | 37 | 36 | 35 | 34 |
|  | Abdomen (T7-12) | 14 | 14 | 14 | 14 | 14 | 12 |
|  | Lumbar and lower limbs (L1-S5) | 8 | 8 | 8 | 8 | 8 | 8 |
|  |  |  |  |  |  |  |  |
