# Supplementary material for: Comparative efficacy of short-term spinal cord stimulation and pulsed radiofrequency in zoster-associated pain: a stratified database study
Source: Front Neurol. 2025 Oct 22;16:1649163. doi: 10.3389/fneur.2025.1649163 (PMC12586037; doi:10.3389/fneur.2025.1649163)
Supplement: Supplementary file 5 [file Table_5.doc]

| **Supplemental Table 5.** Domain-Specific BPI Pain Interference Scores Over Time Between st-SCS and PRF Groups | | | | | | | | |
| --- | --- | --- | --- | --- | --- | --- | --- | --- |
| **Time Point** | **Group** | **GA** | **MD** | **WA** | **NW** | **RO** | **SP** | **EL** |
| baseline | RF group | 6.08(1.74) | 6.18(2.12) | 4.27(1.54) | 5.24(2.34) | 4.69(1.74) | 7.30(1.34) | 6.04(2.41) |
|  | SCS group | 5.96(1.70) | 6.17(1.97) | 4.28(1.57) | 5.16(2.27) | 4.65(1.66) | 7.75(1.29) | 5.81(2.42) |
|  | Z | -0.533 | -0.116 | -0.103 | -0.28 | -0.158 | -1.972 | -0.633 |
|  | *P value* | 0.594 | 0.908 | 0.918 | 0.779 | 0.874 | 0.049^*^ | 0.527 |
| 1m | RF group | 4.46(1.86) | 5.04(2.24) | 2.69(1.65) | 3.72(2.30) | 3.18(1.72) | 6.00(1.63) | 4.49(2.39) |
|  | SCS group | 4.98(1.83) | 3.31(1.88) | 3.27(1.78) | 4.21(2.43) | 2.86(1.79) | 3.94(1.57) | 3.07(2.10) |
|  | Z/F | -1.661 | 4.613 | -2.079 | -1.227 | -1.136 | 238.514 | -3.601 |
|  | *P value* | 0.097 | <0.001^*^ | 0.038^*^ | 0.22 | 0.256 | <0.001^*^ | <0.001^*^ |
| 3m | RF group | 3.55(1.96) | 4.08(2.36) | 1.97(1.60) | 2.86(2.25) | 2.15(1.51) | 4.86(1.89) | 3.50(2.39) |
|  | SCS group | 3.89(1.80) | 2.85(1.53) | 2.10(1.72) | 2.94(2.18) | 1.84(156) | 2.35(1.35) | 2.49(1.77) |
|  | Z/F | -1.448 | -3.363 | -0.417 | -0.326 | -1.491 | 221.363 | -2.586 |
|  | *P value* | 0.148 | 0.001^*^ | 0.677 | 0.744 | 0.136 | ＜0.001^*^ | 0.01^*^ |
| 6m | RF group | 2.64(2.04) | 2.95(2.02) | 1.51(1.13) | 2.07(1.70) | 1.51(1.15) | 3.09(1.72) | 2.55(1.73) |
|  | SCS group | 3.22(1.60) | 2.32(1.47) | 1.78(1.53) | 2.41(1.82) | 1.75(1.46) | 2.31(1.22) | 2.43(1.70) |
|  | Z/F | -2.61 | -1.64 | -0.722 | -1.188 | -0.731 | 23.731 | -0.491 |
|  | *P value* | 0.009^*^ | 0.101 | 0.47 | 0.235 | 0.465 | ＜0.001^*^ | 0.623 |
| 12m | RF group | 2.27(2.01) | 2.55(1.97) | 1.18(1.09) | 1.68(1.61) | 1.19(1.09) | 2.62(1.75) | 2.26(1.61) |
|  | SCS group | 3.01(1.58) | 2.09(1.34) | 1.60(1.49) | 2.19(1.75) | 1.59(1.35) | 2.05(1.31) | 2.17(1.60) |
|  | Z/F | -3.09 | -1.086 | -1.681 | -1.968 | -1.913 | 13.609 | -0.293 |
|  | *P value* | 0.002^*^ | 0.278 | 0.093 | 0.049^*^ | 0.056 | ＜0.001^*^ | 0.77 |
| BPI pain interference scores are presented as mean (SD). * indicate statistically significant differences between the SCS and RF groups. For SP (sleep) comparisons at 1, 3, 6, and 12 months, analysis of covariance (ANCOVA) adjusted for baseline scores was applied, while all other between-group comparisons were conducted using the Mann–Whitney U test. Abbreviations: GA, General Activity; MD, Mood; WA, Walking Ability; NW, Normal Work; RO, Relationship with Others; SP, Sleep; EL, Enjoyment of Life. | | | | | | | | |
